# Bioenzymatic and Chemical Derivatization of Renewable Fatty Acids

**DOI:** 10.3390/biom9100566

**Published:** 2019-10-04

**Authors:** Ravi Kumar Akula, Yong-Uk Kwon

**Affiliations:** 1 Department of Chemistry and Nanoscience, Ewha Womans University, Seoul 03760, Korea; 2 Department of Food Science and Engineering, Ewha Womans University, Seoul 03760, Korea

**Keywords:** ω-amino acid, biocatalysis, biomass, α,ω-dicarboxylic acid, fatty acid, oxidation

## Abstract

In addition to our previous efforts toward bioenzymatic and chemical transformations of ricinoleic acid and oleic acid to their corresponding α,ω-dicarboxylic acids via their ester intermediates driven in *Escherichia coli* cells, several efficient oxidation conditions were investigated and optimized for the conversion of ω-hydroxycarboxylic acids to α,ω-dicarboxylic acids. Pd/C-catalyzed oxidation using NaBH_4_ in a basic aqueous alcohol and Ni(II) salt-catalyzed oxidation using aqueous sodium hypochlorite were considered to be excellent as a hybrid reaction for three successive chemical reactions (hydrogenation, hydrolysis, and oxidation) and an eco-friendly, cost-effective, and practical approach, respectively. Omega-hydroxycarboxylic acids and ω-aminocarboxylic acid were also easily prepared as useful building blocks for plastics or bioactive compounds from the bioenzymatically driven ester intermediate. The scope of the developed synthetic methods can be utilized for large-scale synthesis and various derivatizations.

## 1. Introduction

The development of green chemicals, bioplastics, bioactive compounds, pharmaceuticals, and cosmetics from sustainable biomass is an emerging trend in chemistry and related industries. For example, thermoplastic elastomers (TPEs) that show similar properties as those of plastics and rubbery materials can be used as biodegradable plastics [1]. Among them, thermoplastic polyamides show valuable advantages including improved toughness, strength, and thermal stability. Polyamides also show good biocompatibility in biomedical fields [2]. Due to environmental issues related to the use of petrochemicals and fossil fuels, TPEs can be good alternatives. Alpha, omega-dicarboxylic acids and ω-aminocarboxylic acids of medium length (C7 to C13) have been widely used as intermediates or building blocks for the synthesis of hydraulic fluids, lubricants, pharmaceuticals, plasticizers, and TPEs [3,4,5,6,7]. Alpha, omega-dicarboxylic acids have also been used as precursors or building blocks for the synthesis of interesting bioactive compounds such as inhibitors of CDK4/6-mediated cancer [8], DNA sequence-specific ligands [9], and hydroxyproline analogs showing anti-breast cancer activity [10]. Omega-aminocarboxylic acids including 11-aminoundecanoic acid are useful intermediates for the syntheses of fusidic acid derivatives [11], rolipram-type derivatives [12], and ocotillol-type derivatives [13] with interesting biological activities.

Synthesis of these surrogates has been reported chemically [7] and enzymatically [14]. However, the reported methods are not suitable for environmental aspects, large-scale synthesis, and cost-effectiveness. They also require harsh reaction conditions such as high temperatures and pressures, toxic oxidants, and strong acids. For example, 11-aminoundecanoic acid was made from ricinoleic acid methyl ester using the strong acid HBr at very high temperatures of 450−500 °C [3,15]. Similarly, other synthetic procedures have also been reported with improved yields under harsh reaction conditions [16]. In addition, biotransformation routes using *Candida tropicalis* have also been reported [6]. However, these processes are limited by their low yields and the availability of *C. tropicalis*, which is a known pathogen, and nonane, which is obtained from non-renewable biomass or petrochemical feedstocks.

**Figure 1:**
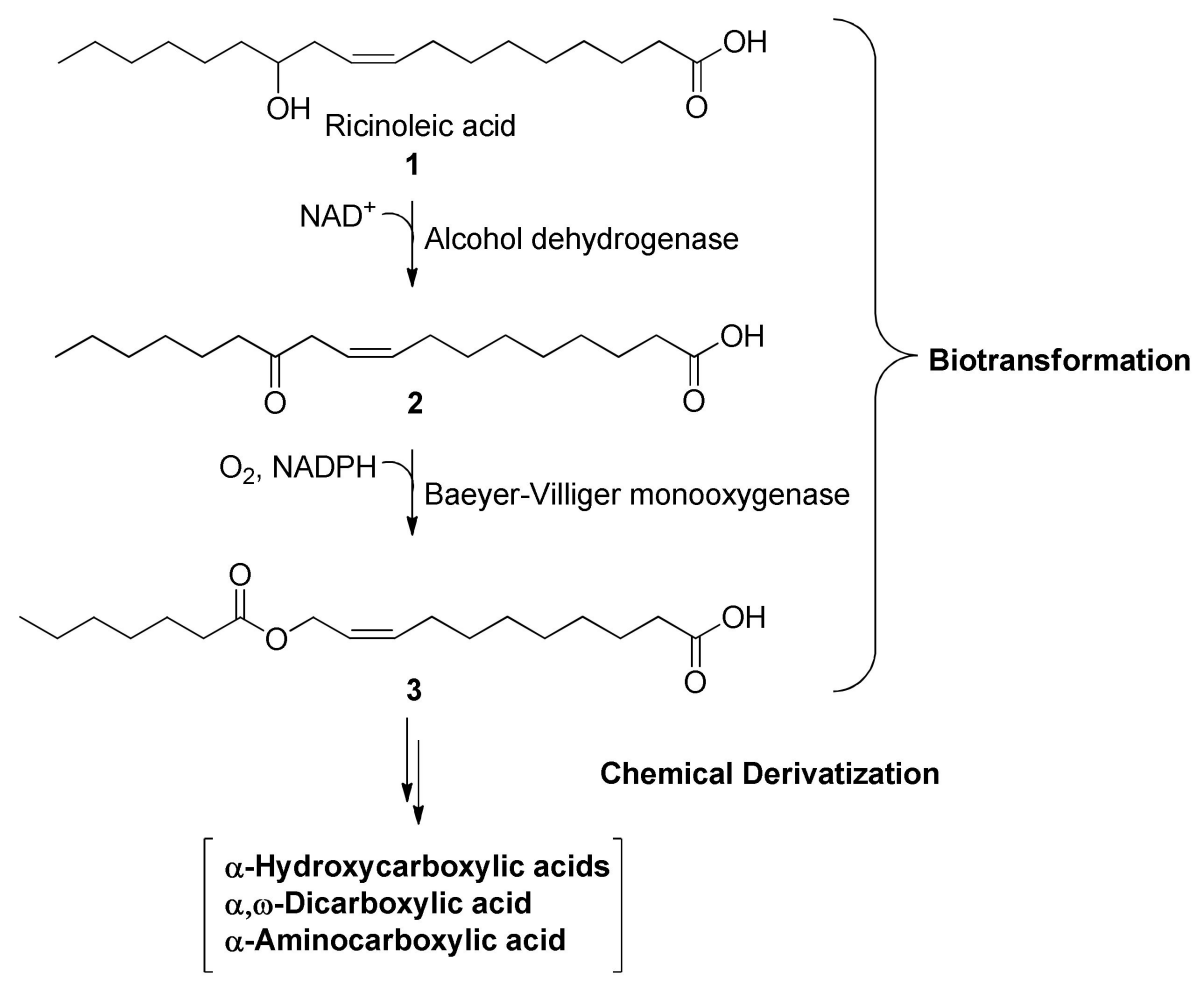
Designed biotransformation pathway of converting ricinoleic acid in *Escherichia coli* cells to ester intermediate (**3**) and its chemical derivatization.

Enzymatic biosyntheses of 1,11-undecanedioic acid, 11-hydroxyundecanoic acid, and 11-aminoundecanoic acid from renewable biomass has been reported [14,17]. Especially, 11-hydroxyundecanoic acid, which is an important intermediate for the synthesis of redox-active naphthoquinones for the treatment of mitochondrial dysfunction [18]. It has also been used as a precursor for the syntheses of acyl-glycine inhibitors of GlyT2 [19] and modulators of RIP2 kinase activity [20]. However, these biosynthetic methods encountered low isolated concentrations of products due to the high toxicity of intermediates or products towards host cells or enzymes. Thus, in order to circumvent this toxicity issue, we recently reported a chemo-enzymatic approach for the synthesis of 11-hydroxyundecanoic acid and 1,11-undecanedioic acid from ricinoleic acid, which is a major fatty acid constituent of castor oil [21]. Biocatalytic transformation of ricinoleic acid into the ester intermediate via 12-ketoolelic acid was achieved in two steps in recombinant *Escherichia coli* cells expressing two essential enzymes: an alcohol dehydrogenase (ADH) from *Micrococcus luteus* and the Baeyer–Villiger monooxygenase (BVMO) from *Pseudomonas putida* KT2440 (Figure 1). Hydrogenation of the olefinic functionality of the ester intermediate followed by alkaline hydrolysis of the ester functional group afforded 11-hydroxyundecanoic acid, which was further oxidized to give 1,11-undecanedioic acid [21]. A similar chemo-enzymatic transformation of oleic acid to azelaic acid (1,9-nonanedioic acid) was successfully demonstrated via the ester intermediate (9-(nonanoyloxy)nonanoic acid) [22]. In addition to our previous efforts, we have envisioned the development of efficient bioenzymatic and chemical strategies for the preparation of α,ω–dicarboxylic acid, ω-hydroxycarboxylic acid, and ω-aminocarboxylic acid from the bioenzymatically driven ester intermediate, which can be used as useful intermediates or building blocks for biodegradable plastics or bioactive compounds as described above. In this study, we aimed to further examine the most efficient, eco-friendly and low-cost oxidation methods of converting ω-hydroxycarboxylic acids to α,ω–dicarboxylic acids, which is still challenging for large-scale synthesis or industrial applications by screening and optimizing several different reaction conditions. Additionally, we tried to develop efficient routes for the derivatization of a bioenzymatically driven ester intermediate to (*Z*)-11-hydroxyundec-9-enoic acid containing an alkenyl functional group and 11-aminoundecanoic acid for various application studies.

## 2. Results and Discussion

### 2.1. Chemical Derivatization of the Bioenzymatically Driven Ester Intermediate

**Scheme 1:**
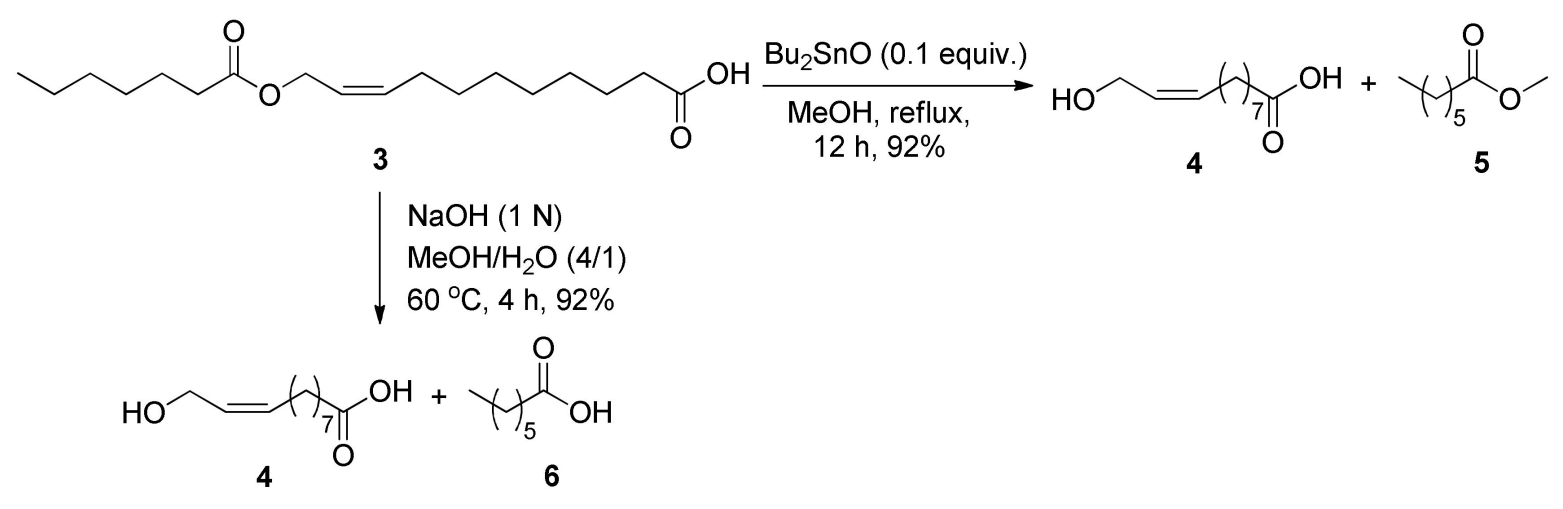
Transesterification and hydrolysis of (*Z*)-11-(heptanoyloxy)undec-9-enoic acid (**3**).

11-Hydroxyundecanoic acid could be synthesized from (*Z*)-11-(heptanoyloxy)undec-9-enoic acid (**3**) [21] or commercially available raw materials such as 10-undecenoic acid [7,23]. In order to enhance the usefulness of the ester intermediate, transesterification of (*Z*)-11-(heptanoyloxy)undec-9-enoic acid (**3**) with methanol in the presence of dibutyltin oxide (Bu_2_SnO) was successfully undertaken to afford (*Z*)-11-hydroxyundec-9-enoic acid (**4**) and methyl heptanoate (**5**) in 92% and 85% yields, respectively [22] (Scheme 1). Interestingly, (*Z*)-11-hydroxyundec-9-enoic acid (**4**) was proven to be a potent antifungal agent isolated from leaves of wild rice, which is resistant to the rice blight fungus [24]. The alkenyl functional group of compound **4** can be further functionalized; dihydroxylation can give diols and oxidative cleavage reactions can provide smaller (nine carbon atoms) fatty acid derivatives. The methyl ester (**5**) can be oxygenated into 7-hydroxy heptanoic acid methyl ester by the alkane monooxygenase AlkBGT from *P. putida* GPo1 [25], which can used as an intermediate for α,ω–dicarboxylic acid and polyesters. 7-Hydroxy heptanoic acid methyl ester can be further converted into 7-aminoheptanoic acid methyl ester by *ω*-transaminase [26], which is an important raw material for the production of several polyamides. The ester intermediate (**3**) could also be hydrolyzed with the hydroxide ion to give (*Z*)-11-hydroxyundec-9-enoic acid (**4**) and heptanoic acid (**6**) in very good yield.

### 2.2. Oxidation of 11-Hydroxyundecanoic Acid to 1,11-Undecanedioic Acid

#### 2.2.1. Optimization for Previously Developed Oxidation Conditions

**Scheme 2:**
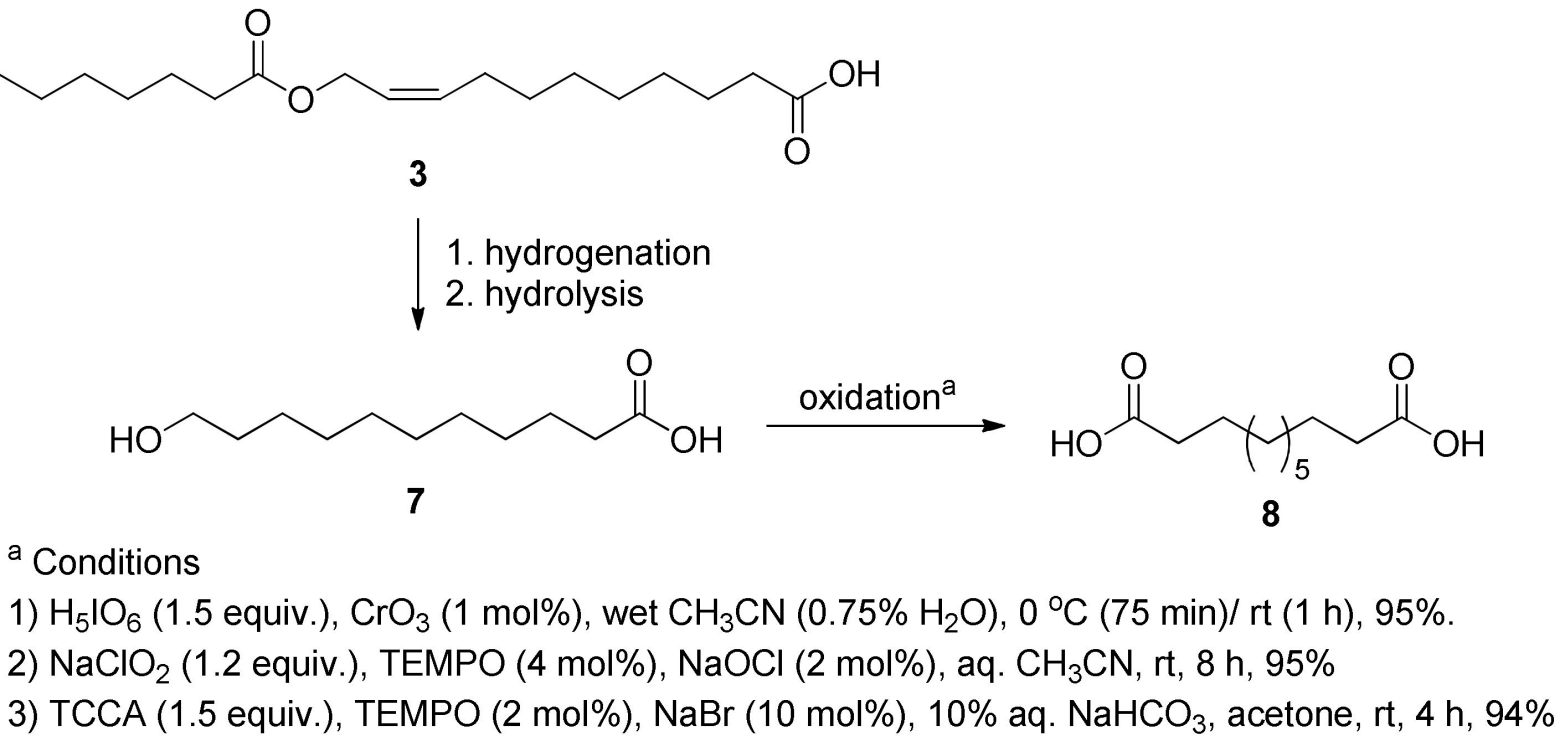
Optimization for previously developed oxidation conditions of 11-hydroxyundecanoic acid (**7**) to 1,11-undecanedioic acid (**8**).

11-Hydroxyundecanoic acid (**7**) was prepared from the ester intermediate, (*Z*)-11-(heptanoyloxy)undec-9-enoic acid (**3**) via a two-step chemical reaction employing hydrogenation with a catalytic amount of Raney^®^-Ni in H_2_ atmosphere and then hydrolysis with NaOH [21]. A previously developed oxidation reaction of 11-hydroxyundecanoic acid (**7**) with periodic acid (H_5_IO_6_) and a catalytic amount of chromium trioxide (CrO_3_) was optimized using H_5_IO_6_ (1.5 equiv.) and CrO_3_ (1 mol%) in wet acetonitrile (CH_3_CN with 0.75% H_2_O) for 75 min at 0 °C and then for 1 h at room temperature (rt) to give an excellent yield (95% yield) of 1,11-undecanedioic acid (**8**) (Scheme 2) [21,22]. Surprisingly, periodic acid-base oxidation conditions in other common solvents such as tetrahydrofuran (THF), 1,4-dioxane, ethyl acetate, acetone, dimethylformamide (DMF), and dimethyl sulfoxide (DMSO) did not work at all.

Other reaction conditions that were applied for the oxidation of 9-hydroxynonanoic acid to azelaic acid [22] were also successfully optimized to oxidize 11-hydroxyundecanoic acid (**7**) to 1,11-undecanedioic acid (**8**) in excellent yields (Scheme 2); 11-hydroxyundecanoic acid (**7**) was treated with NaClO_2_ (1.2 equiv.), (2,2,6,6-tetramethylpiperidin-1-yl)oxyl (TEMPO, 4 mol%) and NaOCl (2 mol%) in aqueous acetonitrile at room temperature (rt) for 8 h to give 1,11-undecanedioic acid (**8**) in 95% yield, and similarly compound **7** was treated with trichloroisocyanuric acid (TCCA, 1.5 equiv.), TEMPO (2 mol%), NaBr (10 mol%) and 10% aqueous NaHCO_3_ in acetone at room temperature for 4 h to give 1,11-undecanedioic acid (**8**) in 94% yield.

#### 2.2.2. Newly Developed Oxidation Methods

For the practical synthesis of 1,11-undecanedioic acid (**8**) via a three-step chemical reaction from the bioenzymatically driven ester intermediate (**3**), environmentally benign, cheap and large scale-suitable oxidation conditions should be developed. Potential methods were thus intensively examined even though previously developed oxidation conditions performed very well. 

At first, the Pd/C-catalyzed aerobic oxidation method was investigated in the presence of an alkaline base, sodium borohydride (NaBH_4_), and an oxygen source in an aqueous alcohol solvent [27]. This method should be interesting in terms of environmental and economical processes. Noxious reagents or acids were not used and the metal catalyst could be reused. Different oxidation conditions were screened to determine an optimal protocol that was slightly modified from the reported procedure (Table 1). For efficient oxidation of 11-hydroxyundecanoic acid (**7**), a nonactivated alkanol, a sufficient amount (6 equiv.) of KOH and a prolonged reaction time were considered important (entries 1−4). In addition, the best results were interestingly obtained when water was finally added to a reaction mixture with pre-activation of the catalytic system for about 10 min (entries 4 and 5). Oxidations with different metal catalysts were also tested; Pt/C gave quite similar results (entries 6 and 7) and the Raney^®^-Ni system did not work at all (entry 8).

Interestingly, some reagents employed in this oxidation were also used for olefin hydrogenation (Pd/C) and ester hydrolysis (KOH) in a three-step conversion of the ester intermediate (**3**) to 1,11-undecanedioic acid (**8**). Thus, a hybrid reaction that involved sequential addition of reagents for the corresponding conversions and final addition of NaBH_4_ (activator of Pd/C) was carried out in a single pot. The hydrogenation and hydrolysis worked very well as expected, but unfortunately the final oxidation did not work: Pd/C might have been deactivated in the two initial steps or might not have been fully activated with NaBH_4_. Sequential reactions in one pot should be time-effective, cost-effective and environmentally benign. Thus, further investigation for the modification or optimization of the reaction conditions and protocol is under progress.

Another oxidation method in green conditions was also examined. A 2-iodobenzoic acid (2-IBAcid)-catalyzed reaction in the presence of Oxone as a co-oxidant was reported for the efficient oxidation of primary and secondary alcohols in aqueous solvents systems (Table 2) [28]. Thus, 11-hydroxyundecanoic acid (**7**) was initially treated with 2-IBAcid (0.3 equiv.) and Oxone (1.3 equiv.) in aqueous acetonitrile, as reported previously, to afford 1,11-undecanedioic acid (**8**) in 76% yield (entry 1). Next, several reactions were performed to determine the optimal oxidation conditions by varying the amount of 2-IBAcid and Oxone used and the reaction time. Compound **7** was oxidized with 0.4 equiv. of 2-IBAcid and 1.6 equiv. of Oxone for 24 h to give 1,11-undecanedioic acid (**8**) in a fairly pure form in excellent yield (entry 4); a lower amount of Oxone was used for a longer time compared to previously used parameters (2.8 equiv. of Oxone and 6 h) for 10-hydroxydecanoic acid. Additionally, oxidation was carried out in water, however, it did not work (entry 5).

Finally, the nickel oxide hydroxide-catalyzed oxidation was examined for an environmentally benign and cheap method (Table 3). Nickel(II) chloride (NiCl_2_) could catalyze the oxidation of primary alcohols with an excess amount of commercial bleach (sodium hypochlorite, NaOCl) to carboxylic acids [29]. At first, 11-hydroxyundecanoic acid (**7**) was oxidized under the reported conditions with 5 mol% of NiCl_2_∙6H_2_O and 9 mL of NaOCl solution (10%) in H_2_O/CH_2_Cl_2_ to give the diacid product (**8**) in 82% yield (entry 1). With a higher amount of NaOCl solution, 11-hydroxyundecanoic acid (**7**) was almost quantitatively oxidized to 1,11-undecanedioic acid (**8**) in a fairly pure form (entry 2). This result should be considered excellent because hexane-1,6-diol has been previously reported to be oxidized to the corresponding diacid in about 70% yield [29]. Oxidation reactions in small volumes of other organic solvents also worked very well (entries 3−5). Interestingly, compound **7** was easily oxidized in only water to the diacid product (**8**) in very good yield (entry 6). Thus, oxidations could be carried out with cheap reagents in aqueous solutions with or without an organic solvent, providing an attractive green strategy with enhanced availability and utility. In addition, these conditions can be applied to a large-scale preparation. Among the oxidation reactions of ω-hydroxycarboxylic acids developed so far, we believe this method is one of the most eco-friendly, cost-effective, and practical approaches to the large-scale synthesis of 1,11-undecanedioic acid (**8**).

So far, currently developed oxidation conditions have also been successfully applied in the oxidation of 9-hydroxynonanoic acid, which was obtained from 9-(nonanoyloxy)nonanoic acid, the ester intermediate in the biotransformation of oleic acid [22]. The development of optimal successive reaction conditions without purification of intermediates via a three-step reaction from the bioenzymatically-driven ester intermediate (**3**) to 1,11-undecanedioic acid (**8**) is still in progress.

### 2.3. Chemical Conversion of 11-Hydroxyundecanoic Acid to 11-Aminoundecanoic Acid

**Scheme 3:**
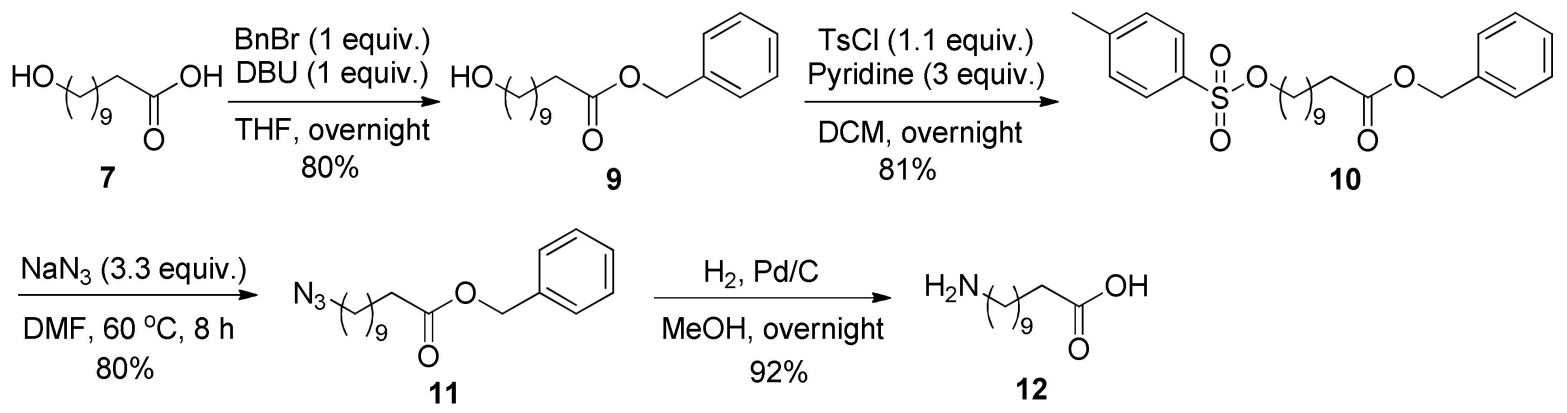
Synthesis of 11-aminoundecanoic acid from 11-hydroxyundecanoic acid.

Several synthetic methods for producing 11-aminoundecanoic acid have been reported but are based on harsh reaction conditions and high temperatures [3,7,15,16]. Thus, we envisioned the efficient synthesis of 11-aminoundecanoic acid from 11-hydroxyundecanoic acid (**7**) using mild reaction conditions (Scheme 3). Due to the polar nature of the target compound, we employed hydrogenation/hydrogenolysis for the deprotection of both amino and carboxylic acid functional groups at the final deprotection step to facilitate easy purification. 

For the conversion of the hydroxyl functional group to an amino functional group, we first protected the carboxylic acid group of compound **7** with a benzyl group. Compound **7** was treated with benzyl bromide (BnBr) in the presence of 1,8-diazabicyclo[5.4.0]undec-7-ene (DBU) in THF overnight at room temperature to afford compound **9** in 80 % yield [30]. Then, compound **9** was treated with *p*-toluenesulfonyl chloride (TsCl) and pyridine in DCM to give the tosylate (**10**) in 81% yield. The tosyl group of compound **10** went through the displacement with sodium azide (NaN_3_) in DMF for 8 h at 60 °C to afford benzyl 11-azidoundecanoate (**11**) showing good conversion [31]. At the final step, both azido and benzyl carboxylate groups of compound **11** were fully deprotected by the treatment with H_2_ in the presence of Pd/C in MeOH overnight at room temperature to afford 11-aminoundecanoic acid (**12**) in 92% yield. Thus, 11-aminoundecanoic acid (**12**) was efficiently prepared from 11-hydroxyundecanoic acid (**7**) in overall 48% yield over four steps.

## 3. Materials and Methods

### 3.1. General Experimental Methods

Chemical reagents were purchased from commercial sources and used without further purification unless noted otherwise. Analytical thin layer chromatography (TLC) was performed on a Merck 60 F_254_ silica gel plate (0.25 mm thickness, Merck KGaA, Darmstadt, Germany), visualization was undertaken with an ultraviolet (UV) light and/or by spraying with a 5% solution of phosphomolybdic acid followed by charring with a heat gun. Flash column chromatography was performed on Merck 60 silica gel (70–230 mesh). Proton nuclear magnetic resonance (^1^H-NMR) and ^13^C-NMR spectra were recorded on a Bruker AVANCE III 3000 (300 MHz, Bruker Korea Co., Ltd., Seongnam-si, Gyeonggi-do, Korea) FT-NMR spectrometer. Chemical shifts were reported in ppm with tetramethylsilane as an internal standard. Matrix-assisted laser desorption/ionization-time of flight (MALDI-TOF) mass analysis was performed using an Axima Performance mass spectrometer (Shimadzu, Manchester, England) with α-cyano-4-hydroxycinnamic acid as a matrix.

### 3.2. Chemical Derivatization of the Bioenzymatically-Driven Ester Intermediate *(**3**)*

#### 3.2.1. Methanolysis of (*Z*)-11-(Heptanoyloxy)undec-9-enoic acid (**3**)

To a solution of the bioenzymatically-driven ester intermediate (**3**) (350 mg, 1.12 mmol) in methanol (8 mL), dibutyltin oxide (28 mg, 0.112 mmol) was added, and the mixture was stirred under reflux for 12 h. After cooling, the mixture was poured into a saturated aqueous sodium bicarbonate (6.0 mL) solution and extracted with ethyl acetate three times. The combined organic layer, which might have contained dibutyltin oxide as a white precipitate, was filtered through a Celite pad, dried over sodium sulfate, filtered, and concentrated in vacuo. Methyl heptanoate (**5**) (136 mg, 85%) was purified by distillation (bp 171 °C, 1 atm) as a colorless liquid and (*Z*)-11-hydroxyundec-9-enoic acid (**4**) (206 mg, 92%) [17] was also purified by column chromatography.

(*Z*)-11-Hydroxyundec-9-enoic acid (**4**) [17]: ^1^H NMR (300 MHz, CDCl_3_) δ 6.67 (broad s, 1H), 5.63−5.48 (m, 2H), 4.19 (d, *J* = 6.0 Hz, 2H), 2.33 (t, *J* = 7.5 Hz, 2H), 2.09−2.02 (m, 2H), 1.65−1.57 (m, 2H), 1.38−1.28 (m, 8H) (Figure S1); ^13^C NMR (75 MHz, CDCl_3_) δ 179.4, 133.1, 128.1, 58.4, 34.0, 29.4, 29.0, 28.9, 28.8, 27.3, 24.6 (Figure S2).

Methyl heptanoate (**5**): bp 171 °C, commercial methyl heptanoate: bp 172−173 °C); ^1^H NMR (300 MHz; CDCl_3_) δ 3.66 (s, 3H), 2.31 (t, *J* = 7.5 Hz, 2H), 1.65−1.55 (m, 2H), 1.36−1.22 (m, 6H), 0.89 (t, *J* = 7.5 Hz, 3H) (Figure S3); ^13^C NMR (75 MHz, CDCl_3_) δ 174.2, 51.3, 34.0, 31.4, 28.8, 24.9, 22.4, 13.9 (Figure S4).

#### 3.2.2. Hydrolysis of (*Z*)-11-(Heptanoyloxy)undec-9-enoic acid (**3**)

To a solution of the bioenzymatically driven ester intermediate (**3**) (1.0 g, 3.2 mmol) in methanol (20 mL), water (5 mL) was added, followed by solid NaOH (1.0 g, 25 mmol). The reaction mixture was then heated for 4 h at 60 °C. The reaction mixture was cooled and the pH of the reaction mixture was adjusted to 2 by the slow addition of dilute HCl (6 N) under ice-cold conditions. The reaction mixture was evaporated to reduce the volume to 5−10 mL (until water started to evaporate). The residual mixture was saturated with NaCl and extracted with ethyl acetate (20 mL) three times. The combined organic layer was washed with brine (10 mL) and water (10 mL), dried over anhydrous Na_2_SO_4_, filtered and evaporated. Column purification (50% ethyl acetate-hexane on silica gel column) of the crude material provided (*Z*)-11-hydroxyundec-9-enoic acid (**4**) (590 mg, 92%).

### 3.3. Oxidation of 11-Hydroxyundecanoic Acid *(**7**)* to 1,11-Undecanedioic Acid *(**8**)*

#### 3.3.1. Pd/C-Catalyzed Oxidation Using NaBH_4_ in Basic Aqueous Alcohol [27]

To a stirred solution of 11-hydroxyundecanoic acid (566 mg, 2.80 mmol), Pd/C (10%, 149 mg, 0.14 mmol) and KOH (941 mg, 16.8 mmol) in methanol (7 mL), NaBH_4_ (10.6 mg, 0.28 mmol) was added in portions over 30 s. After stirring for 10 min at room temperature, water (14 mL) was added. The reaction mixture was vigorously stirred under air for four days at room temperature. Then, the mixture was filtered off through a Celite pad and the filtrate was concentrated. The aqueous layer was acidified with 1 N HCl and extracted with 10% methanol in ethyl acetate (3 × 30 mL). The organic layer was washed with brine, dried over anhydrous MgSO_4_, filtered, and concentrated in vacuo to give a crude compound that was recrystallized with ethyl acetate-hexane to afford 1,11-undecanedioic acid (**8**) (514 mg, 85%) as a white solid. 1,11-undecanedioic acid (**8**) [21]: ^1^H NMR (300 MHz, DMSO-*d*_6_) δ 2.17 (t, *J* = 6.6 Hz, 4H), 1.49−1.44 (m, 4H), 1.23 (m, 10H); ^13^C NMR (75 MHz, DMSO-*d*6) δ 174.9 (2C), 34.1 (2C), 29.2, 29.1 (2C), 29.0 (2C), 24.9 (2C).

#### 3.3.2. 2-Iodobenzoic Acid/Oxone-Mediated Oxidation [28]

To a solution of 11-hydroxyundecanoic acid (202 mg, 1 mmol) in acetonitrile/water (15 mL, 2/1 *v/v*), 2-iodobenzoic acid (100 mg, 0.4 mmol) was added, followed by Oxone (1 g, 1.626 mmol). The mixture was then stirred for 24 h at 70 °C. The reaction mixture was then cooled to below 10 °C and the insoluble hypervalent iodine by-products were filtered off. The resulting mixture was extracted with 10% methanol in dichloromethane (DCM) or ethyl acetate (3 × 20 mL), dried over anhydrous MgSO_4_, filtered, and concentrated in vacuo to afford 1,11-undecanedioic acid (**8**) (209 mg, 97%) in fairly pure form as a white solid. If necessary, the final compound could be further purified by recrystallization.

#### 3.3.3. Nickel(II) Salt-Catalyzed Oxidation Using Aqueous Sodium Hypochlorite [29]

To a stirred solution of NiCl_2_∙6H_2_O (30 mg, 0.12 mmol) in H_2_O (1 mL), 11-hydroxyundecanoic acid (500 mg, 2.47 mmol) and dichloromethane (DCM) (2 mL) were added. Cold aqueous NaOCl (ca. 10%, 18 mL) was added to the reaction mixture over 2 min at 0 °C. The resulting mixture was stirred for 2 h at 0 °C and 2 h at room temperature. The reaction mixture was then treated with 10% Na_2_S_2_O_3_ (10 mL) and stirred for 10 min at room temperature. Then, it was fully acidified with 1 N HCl. The reaction mixture was thoroughly extracted with 10% methanol in DCM or ethyl acetate (3 × 30 mL). The organic layer was washed with brine, dried over anhydrous MgSO_4_, filtered, and concentrated in vacuo to afford 1,11-undecanedioic acid (**8**) (500 mg, 94%) in an almost pure form as a white solid. If necessary, the final compound could be further purified by recrystallization.

### 3.4. Chemical Conversion of 11-Hydroxyundecanoic Acid *(**7**)* to 11-Aminoundecanoic Acid *(**12**)*

#### 3.4.1. Synthesis of Benzyl 11-Hydroxyundecanoate (**9**) [30]

To a solution of 11-hydroxyundecanoic acid (**7**) (700 mg, 3.46 mmol) in tetrahydrofuran (THF) (5 mL) at 0 °C, benzyl bromide (0.41 mL, 3.46 mmol) and 1,8-diazabicyclo[5.4.0]undec-7-ene (DBU) (0.52 mL, 3.46 mmol) were added. The reaction mixture was stirred overnight at room temperature. Then, water was added, and the reaction mixture was extracted with ethyl acetate, dried over Na_2_SO_4_, filtered, and concentrated. The crude residue was purified by silica gel column chromatography to afford benzyl 11-hydroxyundecanoate (**9**) (810 mg, 80 %) [30] as a white solid. ^1^H NMR (300 MHz, CDCl_3_) δ 7.36 (m, 5H), 5.12 (s, 2H), 3.63 (t, *J* = 6.6 Hz, 2H), 2.36 (t, *J* = 7.5 Hz, 2H), 1.92 (s, 1H), 1.54−1.67 (m, 4H), 1.29 (m, 12H) (Figure S5).

#### 3.4.2. Synthesis of Benzyl 11-(Tosyloxy)undecanoate (**10**)

Benzyl 11-hydroxyundecanoate (**10**) (940 mg, 3.2 mmol) was dissolved in DCM (15 mL), and pyridine (0.77 mL, 9.6 mmol) and tosyl chloride (671 mg, 3.52 mmol) were subsequently added at 0 °C. The mixture was stirred overnight at room temperature. Then it was diluted with DCM and quenched with water. The separated organic layer was washed with brine, dried (Na_2_SO_4_), filtered, and concentrated under reduced pressure. The product was purified by column chromatography to afford benzyl 11-(tosyloxy)undecanoate (**10**) (1.16 g, 81%). ^1^H NMR (300 MHz, CDCl_3_) δ 7.79−7.82 (m, 2H), 7.35−7.40 (m, 7H), 5.13 (s, 2H), 4.03 (t, *J* = 6.6 Hz, 2H), 2.46 (s, 3H), 2.36 (t, *J* = 7.5 Hz, 2H), 1.61−1.66 (m, 4H), 1.23−1.31 (m, 12H) (Figure S6); ^13^C NMR (75 MHz, CDCl_3_) δ 173.6, 144.7, 136.2, 133.2, 129.8, 128.5, 128.1, 128.1, 127.9, 70.7, 66.0, 34.3, 29.3, 29.2, 29.2, 29.1, 28.9, 28.8, 25.3, 24.9, 21.6 (Figure S7); MS (MALDI-TOF) *m/z* calculated for C_25_H_34_O_5_SNa [M + Na]^+^ 469.20, found 469.15.

#### 3.4.3. Synthesis of Benzyl 11-Azidoundecanoate (**11**) [31]

Sodium azide (236 mg, 3.63 mmol) was dissolved in DMF (10 mL) and benzyl 11-(tosyloxy)undecanoate (**10**) (500 mg, 1.1 mmol) was added to the solution. The reaction mixture was stirred for 8 h at 60 °C. After cooling to room temperature, H_2_O (10 mL) was added. The reaction mixture was extracted with ethyl acetate (3 × 20 mL). The combined organic layer was washed with cold water (10 mL) and brine (10 mL), dried over Na_2_SO_4_, filtered, and concentrated in vacuo. The product was purified by column chromatography to afford benzyl 11-azidoundecanoate (284 mg, 80%) [31] as a colorless oil. ^1^H NMR (300 MHz, CDCl_3_) δ 7.32−7.37 (m, 5H), 5.13 (s, 2H), 3.25 (t, *J* = 6.0 Hz, 2H), 2.37 (t, *J*=7.5 Hz, 2H), 1.55−1.67 (m, 4H), 1.30−1.36 (m, 12H) (Figure S8); ^13^C NMR (75 MHz, CDCl_3_) δ 173.5, 136.3, 128.5, 128.1, 128.1, 66.0, 51.4, 34.3, 29.4, 29.3, 29.2, 29.13, 29.10, 28.9, 26.7, 24.9 (Figure S9).

#### 3.4.4. Synthesis of 11-Aminoundecanoic Acid (**12**) [7]

To a stirred solution of benzyl 11-azidoundecanoate (**11**) (40 mg, 0.13 mmol) in methanol (4 mL), 10% Pd/C (40 mg) was added and the reaction mixture was stirred in a H_2_ atmosphere (1 atm) overnight at room temperature. Then, the reaction mixture was filtered through a Celite pad and the filtrate was evaporated under reduced pressure to yield 11-aminoundecanoic acid (**12**) (23 mg, 92%) [7]. ^1^H NMR (300 MHz, CD_3_OD) δ 2.81−2.86 (m, 2H), 2.06 (t, *J* = 7.5 Hz, 2H), 1.41−1.52 (m, 4H), 1.19 (s, 12H) (Figure S10).

## 4. Conclusions

The development of green processes for the production of fatty acid derivatives from renewable biomasses is highly desirable. We previously reported on bioenzymatic and chemical transformations of ricinoleic acid and oleic acid via their corresponding ester intermediates driven in *E. coli* cells. In addition to our previous efforts, several efficient oxidation conditions were investigated and optimized in this work, toward the development of eco-friendly, low-cost processes that can be implemented on a large scale. Among them, Pd/C-catalyzed oxidation using NaBH_4_ in a basic aqueous alcohol opened up the possibility that three successive chemical reactions (hydrogenation, hydrolysis and oxidation) could be carried out in a single pot by controlling the addition sequences of reagents. Nickel(II) salt-catalyzed oxidation using aqueous sodium hypochlorite was also revealed to be one of the most eco-friendly, cost-effective, and practical approaches for the large-scale synthesis of α,ω-dicarboxylic acids from ω-hydroxycarboxylic acids. Omega-hydroxycarboxylic acids and ω-aminocarboxylic acid, which can be used as useful building blocks for plastics or bioactive compounds, were also easily prepared from the bioenzymatically driven ester intermediate. 11-Aminoundecanoic acid was efficiently synthesized from 11-hydroxyundecanoic acid in overall 48% yield over four steps. The development of optimal successive reaction conditions for the preparation of α,ω-dicarboxylic acids and further derivatization of the intermediates are still in progress.

## Figures and Tables

**Table 1:**
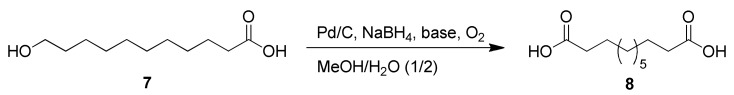
Pd/C-catalyzed oxidation using NaBH_4_ in basic aqueous alcohol.

Entry	Reagent	Temp^a^/Time	Yield (%)
1	Pd/C (10%, 5 mol%), NaBH_4_ (20 mol%), KOH (3 equiv.), O_2_	rt/2 d	n.d.^b^
2	Pd/C (10%, 5 mol%), NaBH_4_ (20 mol%), K_2_CO_3_ (4 equiv.), air	rt/2 d	n.d.^b^
3	Pd/C (10%, 5 mol%), NaBH_4_ (10 mol%), KOH (6 equiv.), air	rt/3 d	71%
4	Pd/C (10%, 5 mol%), NaBH_4_ (10 mol%), KOH (6 equiv.), air	rt/4 d	85%
5	Pd/C (10 %, 5 mol%), NaBH_4_ (10 mol%), KOH (6 equiv.), air	rt/4 d	50%^c^
6	Pt/C (10%, 5 mol%.), NaBH_4_ (10 mol%), KOH (6 equiv.), air	rt/2 d	58%
7	Pt/C (10%, 5 mol%.), NaBH_4_ (10 mol%), KOH (6 equiv.), air	rt/4 d	85%
8	Raney^®^-Ni (5 mol%.), NaBH_4_ (10 mol%), KOH (6 equiv.), air	rt/4 d	n.d.^b^

^a^ rt: room temperature (usually about 25 °C). ^b^ n.d.: not determined, no reaction or very low yield. ^c^ H_2_O was initially added together with CH_3_OH.

**Table 2:**
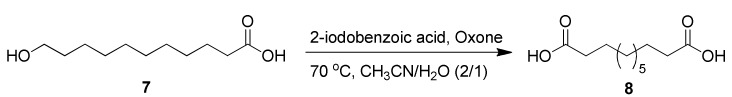
2-Iodobenzoic acid/Oxone-mediated oxidation.

Entry	Reagent	Time (h)	Yield (%)
1	2-IBAcid (0.3 equiv.), Oxone (1.3 equiv.)	6	76%
2	2-IBAcid (0.2 equiv.), Oxone (3.2 equiv.)	9	32%
3	2-IBAcid (0.4 equiv.), Oxone (1.6 equiv.)	9	85%
4	2-IBAcid (0.4 equiv.), Oxone (1.6 equiv.)	24	97%
5	2-IBAcid (0.4 equiv.), Oxone (1.6 equiv.)	9	no reaction^a^

^a^ Only H_2_O was used as a solvent.

**Table 3:**
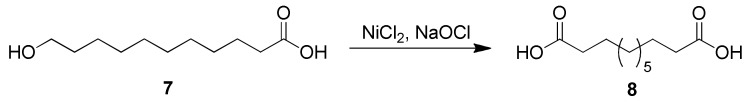
Nickel(II) salt-catalyzed oxidation using aqueous sodium hypochlorite.^a.^

Entry	Solvent^b^	Reagent	Temp/Time	Yield (%)
1	H_2_O/DCM	NiCl_2_ (5 mol%), NaOCl (9 mL)	0 °C/2 h → rt/2 h	82
2	H_2_O/DCM	NiCl_2_ (5 mol%), NaOCl (18 mL)	0 °C/2 h → rt/2 h	94
3	H_2_O/ACN	NiCl_2_ (5 mol%), NaOCl (18 mL)	0 °C/2 h → rt/2 h	94
4	H_2_O/THF	NiCl_2_ (5 mol%), NaOCl (18 mL)	0 °C/2 h → rt/2 h	90
5	H_2_O/1,4-dioxane	NiCl_2_ (5 mol%), NaOCl (18 mL)	0 °C/2 h → rt/2 h	94
6	H_2_O	NiCl_2_ (5 mol%), NaOCl (18 mL)	0 °C/2 h → rt/2 h	85

^a^ 11-Hydroxyundecanoic acid (0.5 g, 2.47 mmol), NiCl_2_∙6H_2_O (5 mol%), NaOCl (ca. 10%), water (1 mL), and organic solvent (2 mL) were used. ^b^ DCM: dichloromethane; ACN: acetonitrile; THF: tetrahydrofuran.

## Supplementary Materials

The following are available online at https://www.mdpi.com/2218-273X/9/10/566/s1, Figure S1: ^1^H NMR spectrum of (*Z*)-11-hydroxyundec-9-enoic acid (**4**); Figure S2: ^13^C NMR spectrum of (*Z*)-11-hydroxyundec-9-enoic acid (**4**); Figure S3: ^1^H NMR spectrum of methyl heptanoate (**5**); Figure S4: ^13^C NMR spectrum of methyl heptanoate (**5**); Figure S5: ^1^H NMR spectrum of benzyl 11-hydroxyundecanoate (**9**); Figure S6: ^1^H NMR spectrum of benzyl 11-(tosyloxy)undecanoate (**10**); Figure S7: ^13^C NMR spectrum of benzyl 11-(tosyloxy)undecanoate (**10**); Figure S8: ^1^H NMR spectrum of benzyl 11-azidoundecanoate (**11**); Figure S9: ^13^C NMR spectrum of Benzyl 11-azidoundecanoate (**11**); Figure S10: ^1^H NMR spectrum of 11-aminoundecanoic acid (**12**).
