# Supplementary material for: Bioenzymatic and Chemical Derivatization of Renewable Fatty Acids
Source: Biomolecules. 2019 Oct 4;9(10):566. doi: 10.3390/biom9100566 (PMC6843907; doi:10.3390/biom9100566)
Supplement: Supplementary file 1 [file biomolecules-09-00566-s001.pdf]

biomolecules

MDPI

Supplementary Materials

Bioenzymatic and Chemical Derivatization of Renewable Fatty Acids

Ravi Kumar Akula[1,2] and Yong-Uk Kwon[1,*]

[1] Department of Chemistry and Nanoscience, Ewha Womans University, Seoul 03760, Republic of Korea
[2] Department of Food Science and Engineering, Ewha Womans University, Seoul 03760, Republic of Korea
* Correspondence: yukwon@ewha.ac.kr; Tel.: +82-2-3277-6685

Table of contents

Figure S1. ^{1}H NMR spectrum of (*Z*)-11-hydroxyundec-9-enoic acid (**4**) ------------------------ S2
Figure S2. ^{13}C NMR spectrum of (*Z*)-11-hydroxyundec-9-enoic acid (**4**) ------------------------ S2
Figure S3. ^{1}H NMR spectrum of methyl heptanoate (**5**) --- S3
Figure S4. ^{13}C NMR spectrum of methyl heptanoate (**5**) --- S3
Figure S5. ^{1}H NMR spectrum of benzyl 11-hydroxyundecanoate (**9**) ------------------------ S4
Figure S6. ^{1}H NMR spectrum of benzyl 11-(tosyloxy)undecanoate (**10**) ------------------------ S4
Figure S7. ^{13}C NMR spectrum of benzyl 11-(tosyloxy)undecanoate (**10**) ------------------------ S5
Figure S8. ^{1}H NMR spectrum of benzyl 11-azidoundecanoate (**11**) ------------------------------ S5
Figure S9. ^{13}C NMR spectrum of Benzyl 11-azidoundecanoate (**11**) ------------------------------ S6
Figure S10. ^{1}H NMR spectrum of 11-aminoundecanoic acid (**12**) ------------------------------ S6

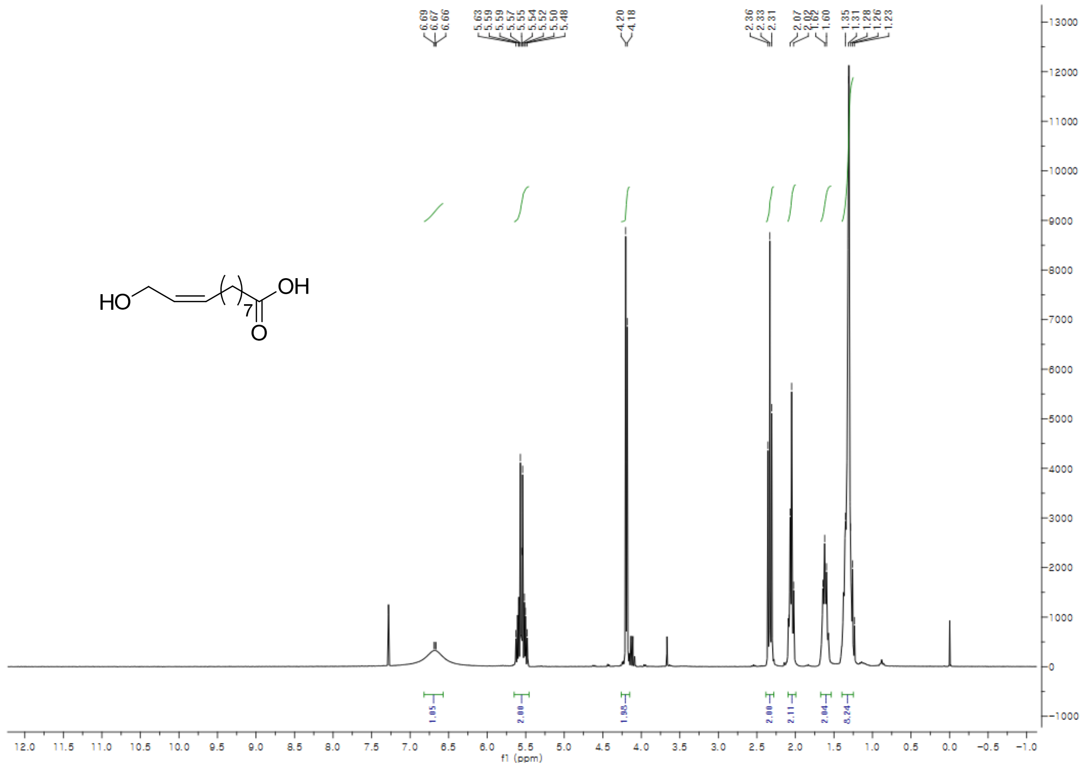

Figure S1. ^{1}H NMR spectrum of (*Z*)-11-hydroxyundec-9-enoic acid (**4**).

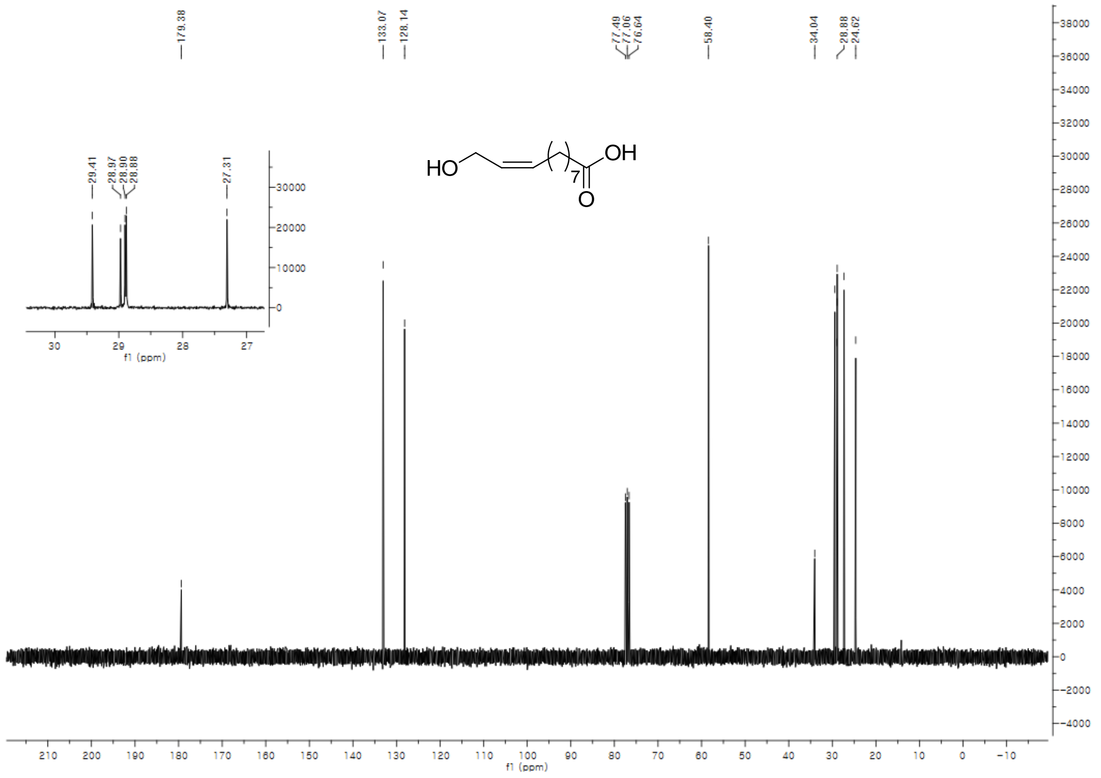

Figure S2. ^{13}C NMR spectrum of (*Z*)-11-hydroxyundec-9-enoic acid (**4**).

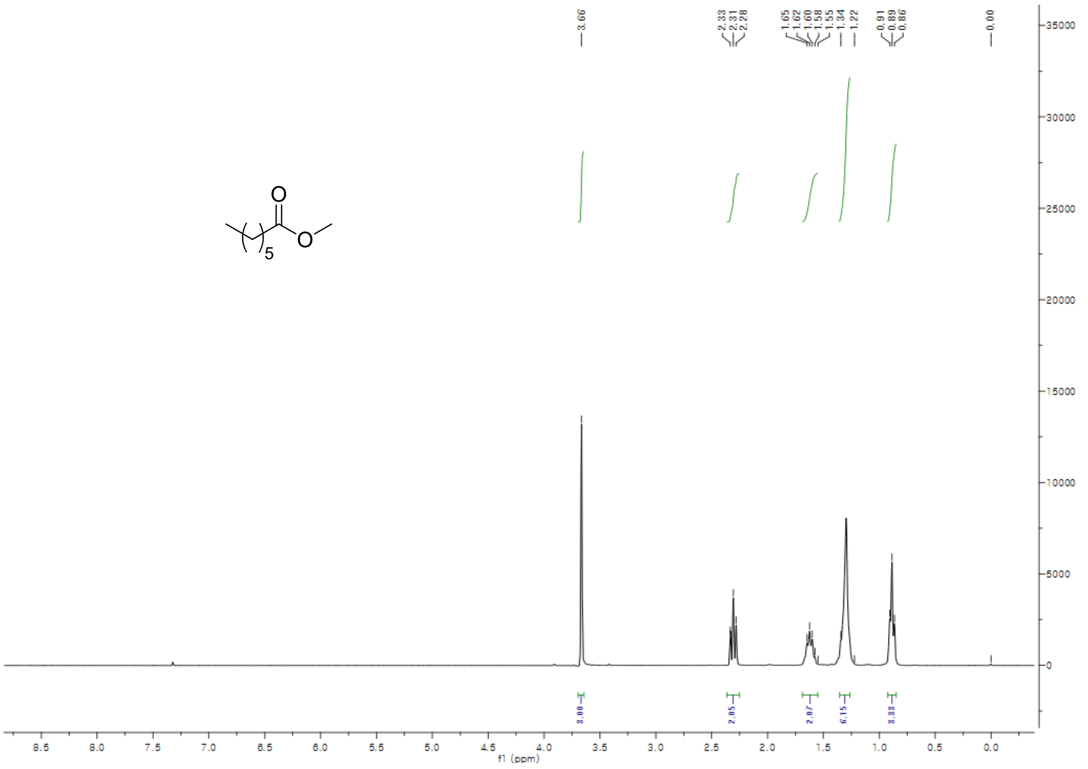

Figure S3. ^{1}H NMR spectrum of methyl heptanoate (**5**).

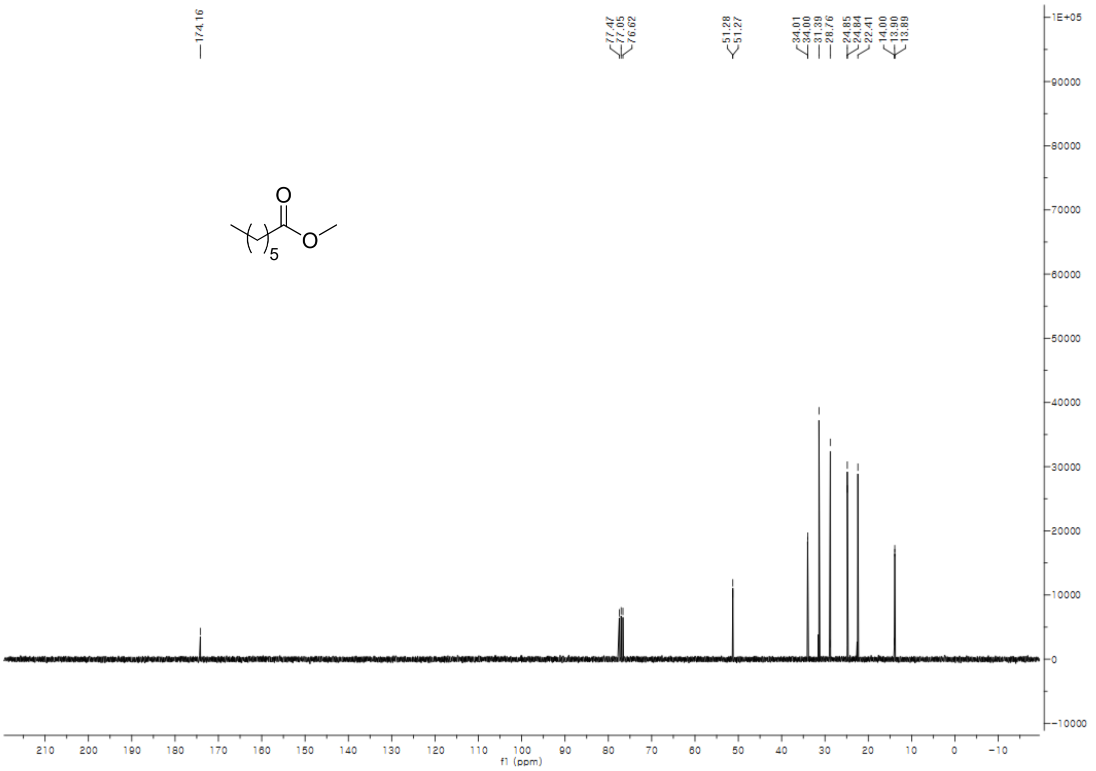

Figure S4. ^{13}C NMR spectrum of methyl heptanoate (**5**).

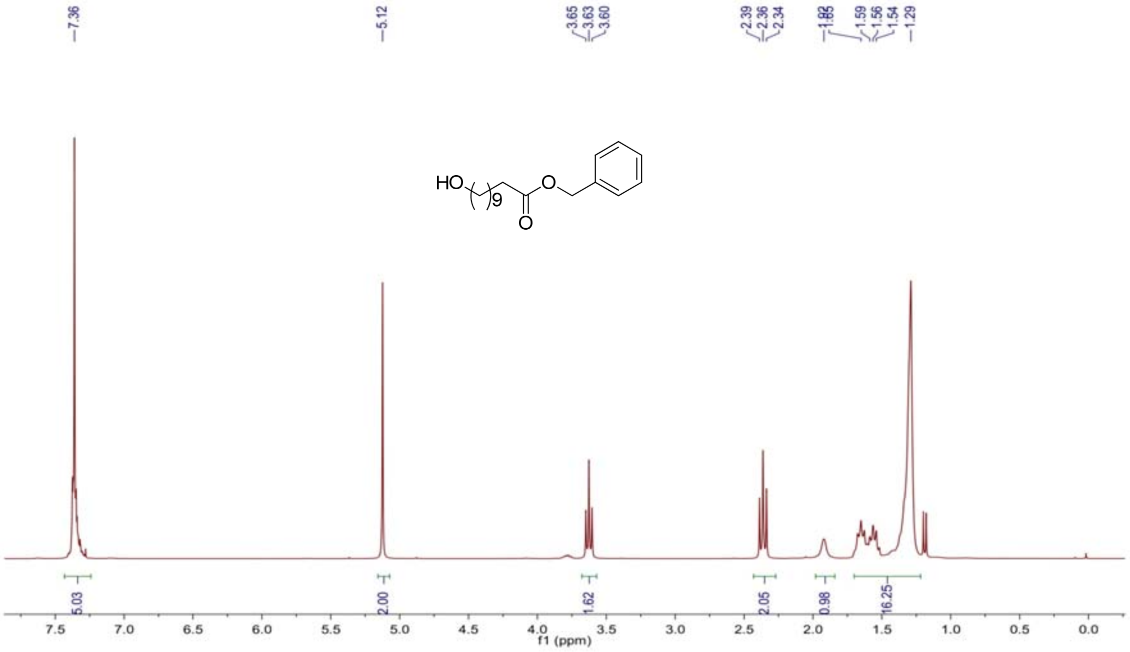

Figure S5. ^{1}H NMR spectrum of benzyl 11-hydroxyundecanoate (**9**).

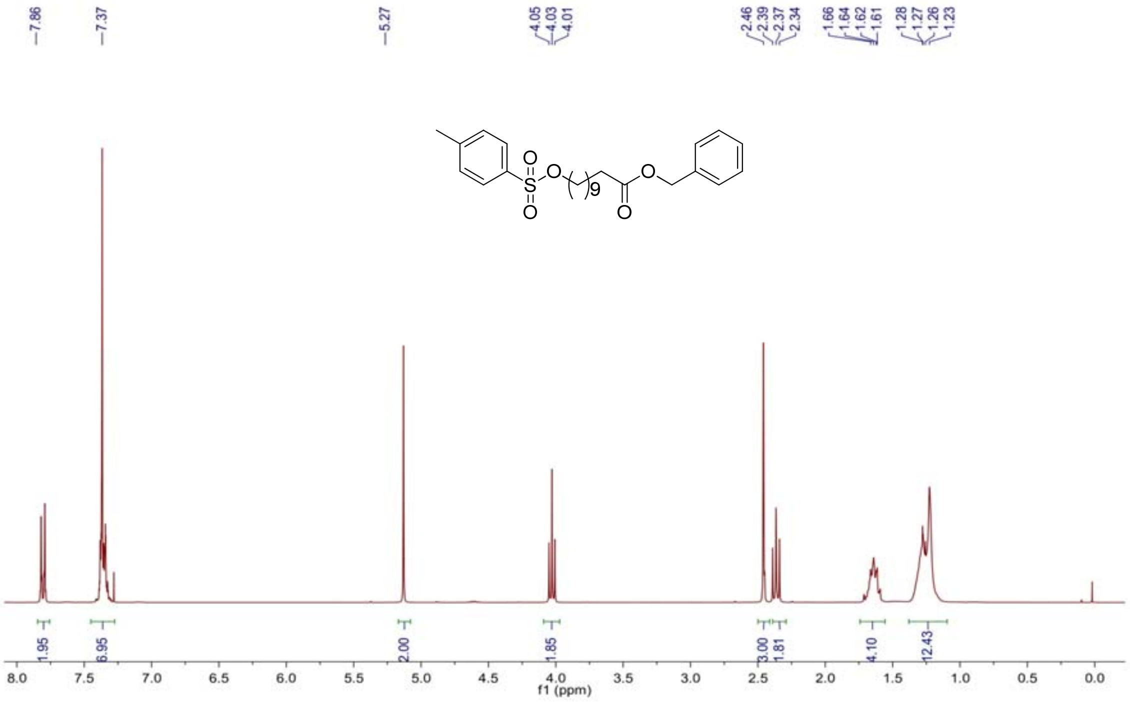

Figure S6. ^{1}H NMR spectrum of benzyl 11-(tosyloxy)undecanoate (**10**).

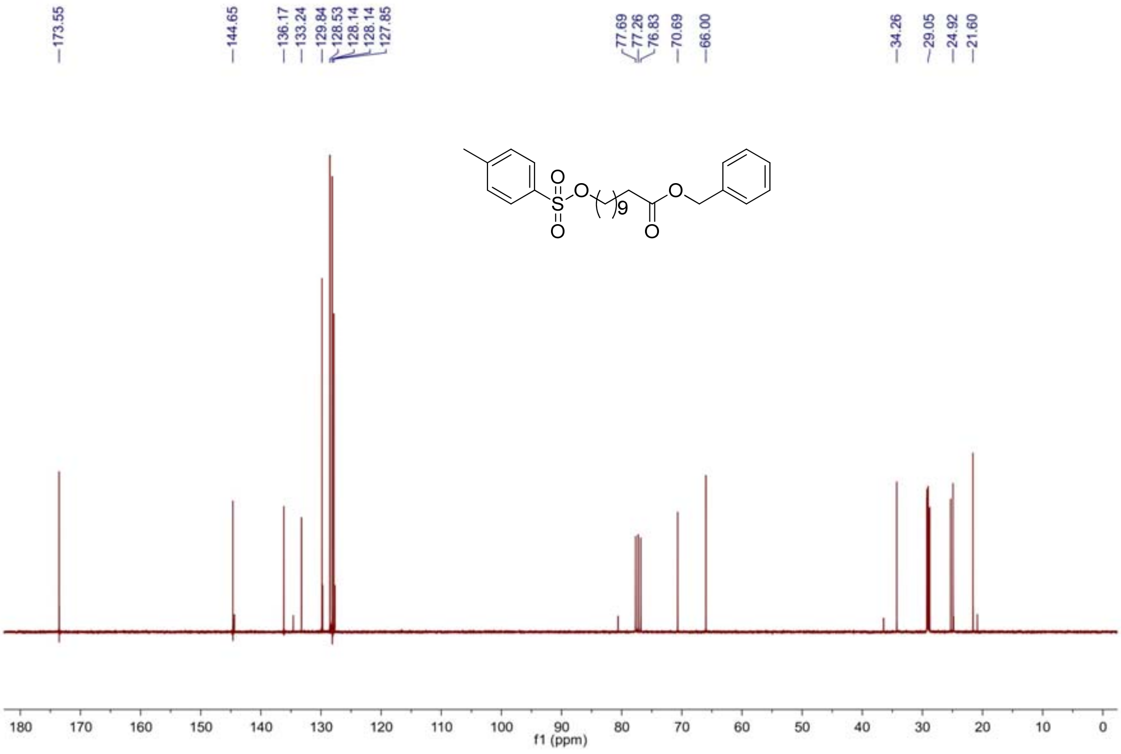

Figure S7. ^{13}C NMR spectrum of benzyl 11-(tosyloxy)undecanoate (**10**).

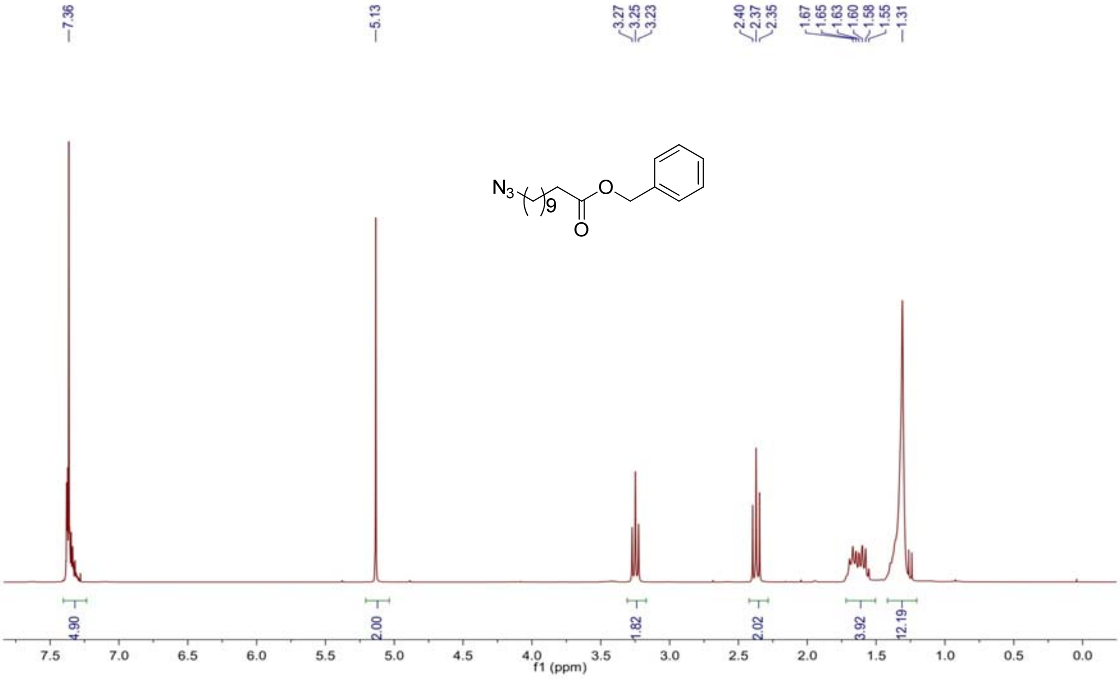

Figure S8. ^{1}H NMR spectrum of benzyl 11-azidoundecanoate (**11**).

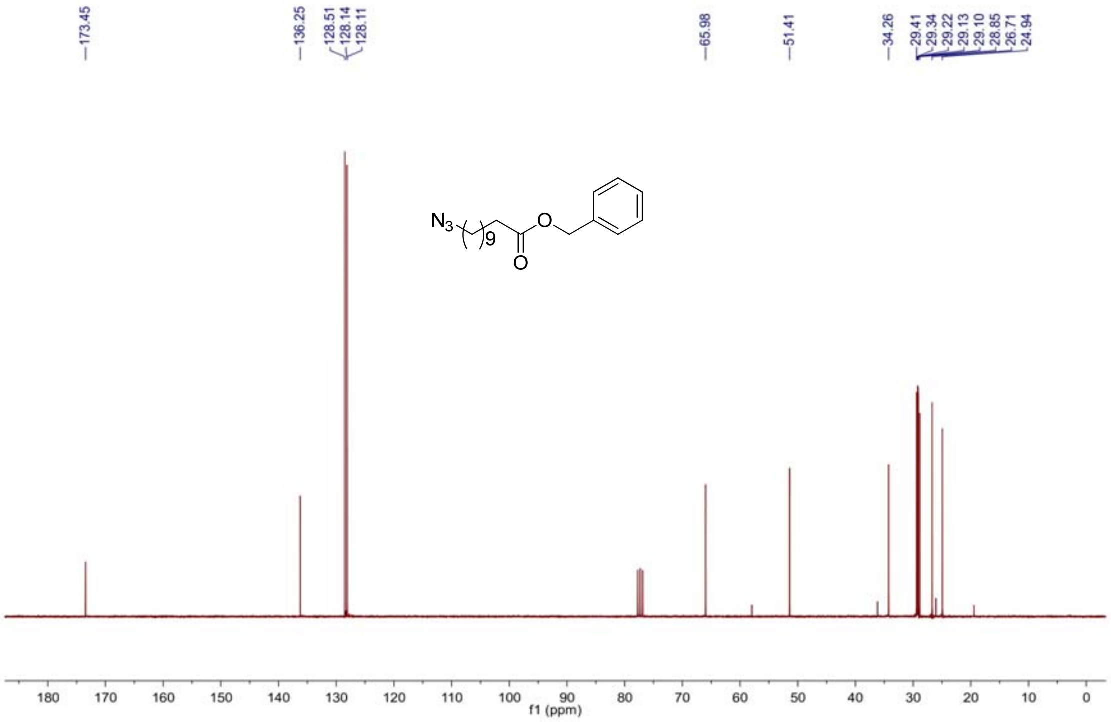

Figure S9. ^{13}C NMR spectrum of Benzyl 11-azidoundecanoate (**11**).

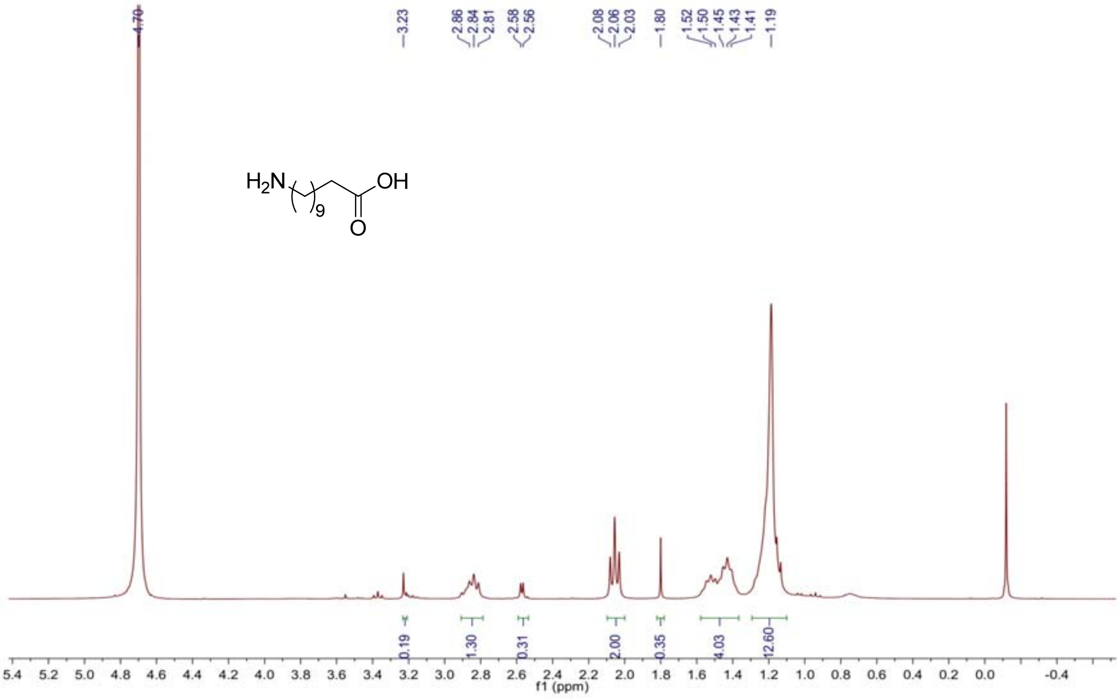

Figure S10. ^{1}H NMR spectrum of 11-aminoundecanoic acid (**12**).
